# Toll-Like Receptor Ligand-Induced Liver Injury in D-Galactosamine-Sensitized Mice: Differences between TLR7/8 and TLR9 Ligands, Cytokine Patterns, and Cross-Tolerance Induction by TLR2 Ligand Pretreatment

**DOI:** 10.1155/2017/9653793

**Published:** 2017-10-17

**Authors:** Reiko Seki

**Affiliations:** Teikyo University School of Medical Technology, Itabashi, Tokyo, Japan

## Abstract

Administration of Toll-like receptor ligands (TLRLs) is known to cause liver injury in D-galN-sensitized mice. In the present study, we aimed to complement preceding reports on the TLRL/D-galN system by analyzing comparisons among TLRLs, mouse strain dependence, effects on serum levels of cytokines, and effects of sequential administrations of different TLRLs. In a preliminary set of analyses, we first confirmed that liver failure can be induced by diverse TLRLs, including LTA and R848 in combination with D-galN. Analysis using TLR4-deficient mice excluded potential confounding effects of endogenous TLR4Ls that include those referred to as DAMPs in CpG DNA/D-galN hepatotoxicity. Subsequently, we showed that LTA pretreatment could prevent mortality in both CpG DNA/D-galN- and R848/D-galN-treated mice compared to without pretreatment. Incidentally, we observed that without the LTA pretreatment, CpG DNA/D-galN showed relatively higher liver-specific toxicity whereas R848/D-galN showed more symptoms of multiple organ failure. These findings suggest that, in D-galN-sensitized mice, different TLRLs not only show similarity in the ability to induce hepatic injury but also exhibit distinctive abilities in inducing systemic inflammation and multiple organ failure. These findings also suggest the potential usefulness of cross-tolerance induction using LTA in the prevention of organ failure in TLRL-mediated acute inflammation.

## 1. Introduction

Experimental models of inflammation-mediated organ failure and sepsis have greatly contributed to our understanding of pathophysiology in such clinical challenges. In an attempt to cope with the relative insensitivity of rodents to lipopolysaccharide (LPS), Galanos et al. [1] found that D-galactosamine (D-galN) dramatically sensitizes mice to LPS. LPS/D-galN administration has been widely used as a model for sepsis, based on the knowledge that LPS induces sepsis. Recently, several lines of studies have established that LPS/D-galN challenge shows development of acute hepatic inflammation often called “fulminant hepatitis” [2], with some reports arguing differences between LPS-induced systemic inflammation and the hepatic injury induced by LPS/D-galN [3]. Tumor necrosis factor-*α* (TNF-*α*) has been shown to be a key mediator in the LPS/D-galN system, causing hepatic apoptosis and subsequent necrosis [4].

D-galN is likely to exert hepatotoxicity by inhibiting the biosynthesis of RNA, proteins, and glycogen in hepatocytes [5]. Besides LPS (ligand for Toll-like receptor 4, TLR4), D-galN-sensitized mice have been shown to exhibit increased sensitivities to the toxic effects of other TLR ligands (TLRLs) including CpG DNA (CpG) (for TLR9) and poly (I:C) (for TLR3) as well as unpurified microbial products [2, 6, 7].

An innate immune response leads to intense long-lasting inflammation and often leads to an adaptive immune response. TLR activation has been utilized for vaccine adjuvants [8], signifying the primary role for TLRs as an enhancer of the immune response. It has been well documented that TLRLs can induce type I interferon (IFN) (e.g., IFN*α*/*β*) and proinflammatory cytokines including IL-1*β*, TNF-*α*, and IL-6 [8]. Activation of TLRs can also induce T helper 1 (T_h_1) cell-inducing cytokines (e.g., IL-12), regulatory T (T_reg_) cell-inducing cytokines (e.g., IL-10), and cytokines which promote the development of T_h_17 cell such as IL-6, transforming growth factor-*β* (TGF-*β*), and IL-23, reflecting essential roles for TLRs in maintaining the balance in T cell response [9].

Apart from induction of adaptive immunity, TLRs have been shown to be involved in broader critical conditions such as ischemia-reperfusion, which may lead to multiple organ failure [10, 11]. Inhibition of TLR4 activity reduced mortality in a mouse model of myocardial infarction and reperfusion injury and reduced inflammatory markers [12]. TLR4 is considered to mediate sepsis-induced acute kidney injury by causing cytokine and chemokine release [13]. However, specific cytokines that mediate such effects of TLR activation on organ injuries are not fully understood. In a mouse injury model caused by liver ischemia-reperfusion, it has been shown that endoplasmic reticulum stress in Kupffer cells induces IL-6 production for the conversion of natural Tregs (nTreg) to T_h_17, which leads to severer liver injury [11].

Another topic in innate immunity that has drawn the interest of researchers is the effects of multiple or sequential TLR stimulations. Some cases of multiple stimulations of TLRs show synergy; however, in many cases of sequential activations of TLRs, an induction of tolerance has been observed [14–16]. In the case of cross-tolerance of TLRLs, after sequential activations of different TLRLs, reduced inflammatory responses are often observed upon the second TLR stimulation by attenuated activation of immune cells involving macrophages. Well-studied examples for tolerance-inducing TLRs involve TLR4 and TLR2. For example, macrophages exposed to TLR2 ligands (TLR2Ls) become hyporesponsive to subsequent stimulation with LPS and vice versa [17]. From a practical perspective, it is of interest whether such tolerance induction by TLR stimulations shows any effects in models of organ injury. It is unknown whether conditions causing intense cell stress such as ischemia-reperfusion or exposure to toxic substances that sensitize cells/tissues/organs exhibit enhanced responses to TLR signaling.

In the present study, an intraperitoneal injection mouse model similar to those used in previous reports was used to address the following specific points. First, to compare different TLRLs including R848 (TLR7/8L) and LTA (TLR2L) in combination with D-galN, we conducted a preliminary set of experiments regarding the induction of hepatic injury. Next, to address the issue of potential influences of endogenous TLR4 ligands, we compared the CpG/D-galN effects between TLR4-deficient and wild-type mice. We think that this is a nontrivial attempt as regulated acetylation and localization of high mobility group box 1 protein (HMGB1), which is known to be a damage-associated molecular pattern (DAMP) and an endogenous TLR4L, has recently been implicated in LPS/D-galN-induced liver failure [18]. Finally, to gain insights into the cytokine patterns and potential cross-tolerance, the effect of LTA pretreatment was examined in CpG/D-galN and R848/D-galN mice. We also discuss our findings that, in non-LTA-pretreated mice, CpG/D-galN and R848/D-galN treatments both caused hepatic injury, however, led to distinct cytokine patterns, distinct strain dependency, and distinct degrees of systemic inflammation.

## 2. Material and Methods

### 2.1. Mice

Five-week-old female inbred C3H/HeN (harboring wild-type TLR4 and simply referred to as C3H), C3H/HeJ (TLR4-deficient), and C57BL/6 mice were purchased from Charles River Japan (Kanagawa, Japan) and acclimated for seven days before the experiment. Mice were housed individually under controlled lighting (with light on from 8 : 00 to 20 : 00 h), temperature (24 ± 0.5°C), humidity (50 ± 10%), and ventilation (~35 complete air exchanges per hour). Food (CRF1; Charles River Japan, Kanagawa, Japan) and water were made available ad libitum.

### 2.2. Reagents

R848 (imidazoquinoline compound), LTA-SA (lipoteichoic acid from *Staphylococcus aureus*), and CpG DNA type B 1826 (5′-CCATGACGTTCCTGACGTT-3′) were purchased from InvivoGen (San Diego, CA, USA). LPS (from *Escherichia coli* 055: B5), poly (I:C) (polyinosinic-polycytidylic acid sodium salt), and D-galN (D-(+)-galactosamine hydrochloride) were purchased from Sigma (Saint Louis, MO, USA).

### 2.3. Experimental Protocol

Six- to seven-week-old C3H/HeN (TLR4(+)), C3H/HeJ (TLR4(−)), and C57BL/6 mice were injected intraperitoneally with PBS of 200 *μ*l per mouse or PBS buffer containing each of the TLRLs and 20 mg/mouse D-galN. Blood was collected at 1 and 5 h, and the mice were sacrificed at 10 h after the injection of TLRLs/D-galN. Immediately after sacrifice, serum was prepared and stored at −80°C until analysis. The serum sample was analyzed for serum alanine aminotransferase (ALT) activity with DRI-CHEM4000 (Fujifilm, Tokyo, Japan) based on the protocol provided by the manufacturer. Serum cytokines (TNF-*α*, IL-6, IFN-*γ*, IL-10, IL-17A, IL-23, and IL-27) were measured by Bio-Plex assay (VERITAS, Tokyo, Japan). All animal experiments were performed in accordance with protocols approved by the experimental animal committee of Teikyo University.

### 2.4. Statistical Analysis

Statistical significance among groups was assessed using the Wilcoxon matched paired-rank test. Survival studies were analyzed with the Kaplan-Meier curve and log-rank test. All analyses were performed using JMP version 12 software (SAS Institute Japan Ltd., Tokyo, Japan). Data in graphs are presented as mean ± SD. *P* values smaller than 0.05 were considered to be significant.

## 3. Results and Discussion

### 3.1. Diverse TLRLs Induce Acute Liver Injury in D-galN-Sensitized Mice

The TLRL/D-galN system has widely been utilized in studies on hepatitis and particularly in evaluating compounds for possible protection from hepatitis. Here, we aimed to complement previous reports in terms of types of TLRLs and strain dependence. Serum ALT levels of the C57BL/6 and C3H mice after intraperitoneal injection of several TLRL/D-galN combinations were measured (Table 1). For all combinations, the dose of D-galN was fixed at 20 mg/mouse. Considering the practical limitation of the total number of mice tested, we acknowledge that this is a pilot study on a small scale (*n* = 3), which should be regarded as a qualitative, not quantitative, trial.

Neither TLRL nor D-galN alone induced an increase in serum ALT levels in both strains (Table 1), in agreement with previous reports [6, 19]. On the other hand, in C57BL/6 mice, all of the TLRLs tested (LPS, LTA, R848, poly (I:C), and CpG) in combination with D-galN resulted in development of severe acute liver injury at high doses. In contrast, in C3H mice, the administration of LPS (TLR4L)/D-galN and R848 (TLR7/8L)/D-galN caused as severe lethal hepatic injury as in the C57BL/6 mice; however, for the remaining TLRLs (i.e., LTA (TLR2L), poly (I:C) (TLR3L), and CpG (TLR9L)), administration in combination with D-galN induced only modest degrees of increased serum ALT. Although this small-scale analysis did not allow us to draw any significant conclusions, it raised the possibility that there is strain dependency in sensitivity to LTA, poly (I:C), and CpG in D-galN-sensitized mice.

The results presented in Table 1 confirm previous findings on the toxicities of LPS/D-galN, poly (I:C)/D-galN, and CpG/D-galN [4, 6, 19]. Although this was a qualitative analysis, LTA (TLR2L) and R848 (TLR7/8L), which have not previously been well studied in this setting, were shown to induce hepatic injury in D-galN-sensitized mice. To confirm the strain dependency suggested in the above (i.e., sensitivity of C57BL/6 but not C3H mice to ligands including CpG/D-galN and sensitivity of both strains to those including R848/D-galN) and to further address the difference between these TLRLs in the pattern of serum proinflammatory cytokines, we added another set of experiments that compared C57BL/6 and C3H mice as well as CpG and R848 (*n* = 8). The strain dependency was replicated based on the ALT value after the stimulation for both TLRLs in D-galN-sensitized mice. Indeed, here again, C3H was largely insensitive to CpG/D-galN and sensitive to R848/D-galN, whereas C57BL/6 was sensitive to both (Table 2). Both TNF-*α* and IL-6 values showed correlations with the ALT values, in line with the previous finding that TNF-*α* is the key cytokine mediating the hepatic toxicity of the TLRLs/D-galN [7]. These data confirm the previous finding that CpG/D-galN treatment causes increases in these cytokines, suggesting key roles for these cytokines in hepatotoxicity [6]. These data also showed that R848/D-galN treatment causes increases in these proinflammatory cytokines, suggesting that these are likely to be key mediators for the hepatotoxicity of R848/D-galN.

Intriguingly, despite the high mortality of the mice treated with R848/D-galN (all eight mice died within 9 h after injection for both strains) relative to the CpG/D-galN-treated mice (only four and none of eight died within 10 h for C57BL/6 and C3H mice, resp.), none of the values of ALT, TNF-*α*, or IL-6 showed a good correlation with the survival data (Table 2). Another feature of these findings was that the responses to CpG/D-galN, but not to R848/D-galN, showed strain dependency. Overall, these findings suggested that the levels of TNF-*α* and IL-6 have good prognostic value for hepatic injury as well as the survival rate for CpG/D-galN-treated mice; however, R848/D-galN-treated mice tended to show high mortality despite relatively modest increases in these cytokine levels.

### 3.2. TLR4 Is Not Involved in CpG/D-galN-Induced Liver Injury

**Figure 1:**
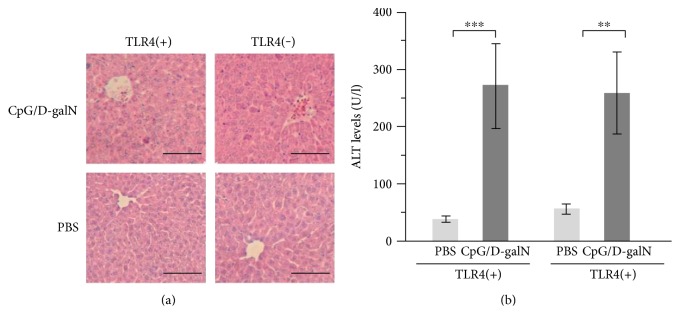
Effect of CpG/D-galN in TLR4(+) and (−) mice. (a) Histological analysis of the liver with hematoxylin-eosin staining. Scale bars indicate 100 *μ*m. (b) Serum ALT levels. ^∗∗^*p* < 0.05; ^∗∗∗^*p* < 0.03.

In the above shown was the ability of various TLRLs combined with D-galN to induce liver injury (Tables 1 and 2); however, to the best of our knowledge, no report has formally ruled out the possibility that inflammation is amplified by endogenous ligands for TLR4 including DAMPs in mice treated with TLRLs/D-galN. This is important, as acetylation and localization of HMGB1, a DAMP and an endogenous TLR4 ligand, have recently been shown to undergo changes in LPS/D-galN-induced liver failure [19]. CpG/D-galN can give rise to HMGB1 and heat shock proteins, both being endogenous DAMPs that activate TLR4 [21]. Given these findings, we compared C3H/HeJ (TLR4 deficient) strain and C3H/HeN strain that harbors wild-type TLR4 (TLR4(+)) and the effects of CpG/D-galN treatment (*n* = 7). The C3H/HeJ (TLR4(−)) mice exhibited a similar degree of liver injury as in C3H/HeN (TLR4(+)) mice at 10 h after the injection (Figure 1). On histological examination, for both strains, the CpG/D-galN-treated mice, but not PBS-injected mice, showed numerous inflammatory foci in the pericentral area with signs of inflammatory liver damage, based on observations of hemorrhage, infiltration of lymphocytes, and scattered eosinophilic degenerative damages of liver cells, which were in good agreement with the similar analysis by Yi et al. [6]. The average of the serum ALT levels in the CpG/D-galN-treated TLR4(+) mice (267.7 IU/l) was not different from that in TLR4(−) mice (258.4 IU/l). Therefore, although we did not test for other TLRLs, these results suggest that the possible confounding effects of endogenous TLR4Ls on TLR9L/D-galN-induced hepatic injury are negligible. The results also rule out a substantial effect of translocation of components of commensal bacteria into the circulation, which could provide LPS.

### 3.3. LTA Pretreatment Protects Mice from Lethal Liver Injury by CpG/D-galN as well as by R848/D-galN, Whereas These Combinations Differ in Liver versus Systemic Toxicity

**Figure 2:**
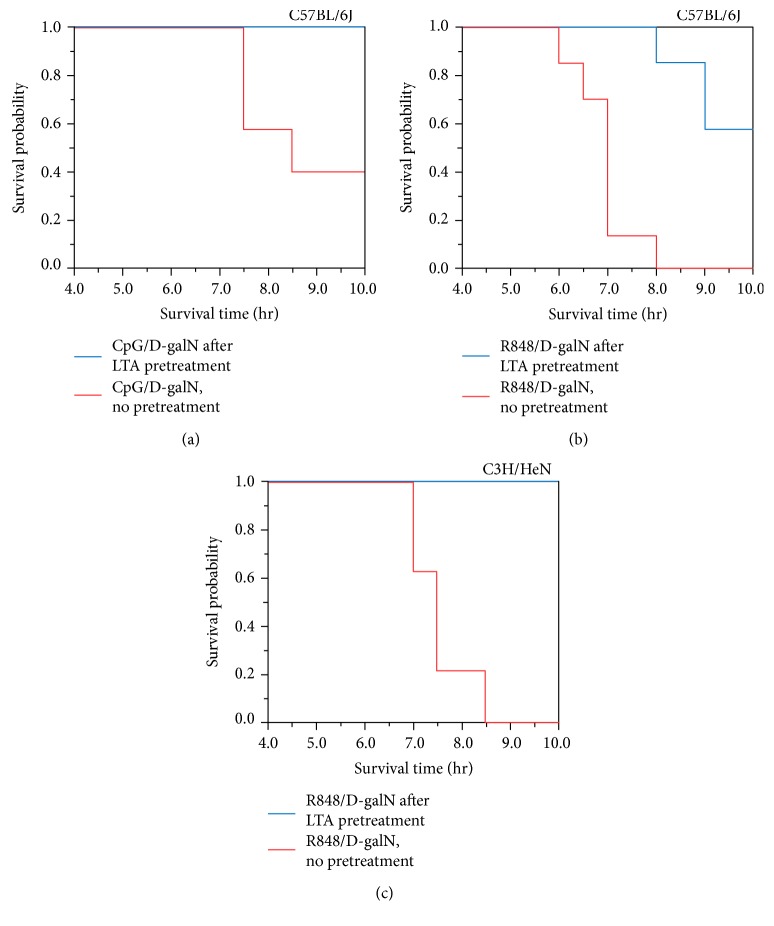
Survival rate analysis of the tolerance-inducing effect of LTA pretreatment based on the Kaplan-Meier method. (a) C57BL/6J mice treated with CpG/D-galN. (b) C57BL/6J mice treated with R848/D-galN. (c) C3H mice treated with R848/D-galN.

Experiments with sequential stimulations have shown that pretreatment with a TLRL can lead to priming (enhancement) or attenuation of effects of the secondary TLR challenge. In particular, LTA (TLR2L) can induce cross-tolerance against a second challenge with LPS [17, 22, 23]. To examine the effects of a TLR2L pretreatment on the liver injury caused by TLRL/D-galN, we administered CpG/D-galN to C57BL/6 mice without or after LTA pretreatment. Without LTA pretreatment (200 *μ*g/mouse), four of seven mice died within 9 h after the CpG/D-galN challenge (Figure 2(a)), which is consistent with the above results. Mortality was significantly reduced in LTA-pretreated mice (Figure 2(a)). When R848/D-galN was used for the second injection, a similar improvement in survival was observed (Figure 2(b)). When C3H instead of C57BL/6 mice were used, a similar improvement in the survival rate was observed in the mice with the LTA pretreatment prior to the R848/D-galN administration (Figure 2(c)). These findings indicate that LTA pretreatment can induce cross-tolerance against the second challenge with either CpG/D-galN or R848/D-galN.

Interestingly, during the experiments without LTA pretreatment, it was observed that all R848/D-galN mice exhibited severe signs of illness (immobility, rough fur, massive blood coagulation, gastrointestinal bleeding, and decrease in body temperature) whereas CpG/D-galN mice did not show any of these symptoms. Moreover, all of the R848/D-galN mice died 6–9 h after the challenge whereas four out of eight CpG/GalN mice survived at 10 h. Analysis of several organs/tissues of the dead R848/GalN mice showed features consistent with multiple organ failure, whereas none of the CpG/D-galN mice showed these symptoms (data not shown).

To gain further insight into the difference between the two TLRLs (CpG and R848) and the effect of LTA pretreatment on the toxicity of these TLRL/D-galN treatments, serum cytokine levels were measured using C57BL/6 mice. The cytokines measured here more or less focused on those associated with Th17 cells (IL-17A, IL-23, and IL-27) based on recent general interest in a Th17 in ischemic-reperfusion and sepsis models [11] as well as in an LPS/D-galN hepatic injury model [24].

Without LTA pretreatment, the two sets of mice (CpG/D-galN and R848/D-galN) showed similar cytokine patterns although it was difficult to draw conclusive differences between the R848 and CpG in our experimental setting (Table 3). One common feature was the induction of IL-6 and TNF-*α*. Contrary to our anticipation, the cytokines important for Th17 (IL-17A, IL-23, and IL-27) did not exhibit appreciable changes for both combinations of TLRL/D-galN. Another intriguing feature was relatively high IL-10 levels in the CpG/D-galN-treated mice compared to that in the R848/D-galN-treated mice (Table 3).

Conversely, the CpG/D-galN-treated mice exhibited clear suppression of IL-6 and TNF-*α* inductions after LTA pretreatment. Intriguingly, the R848/D-galN-treated mice did not exhibit clear suppression of IL-6 and TNF-*α* inductions by LTA pretreatment. For both CpG/D-galN and R848/D-galN, LTA-induced tolerance (Figure 2) was not accompanied by an increase in IL-10 levels, which was rather shown to decrease (Table 3). Although the decrease in IL-10 levels and increases in IL-17A, IL-23, and IL-27 levels were notable (Table 3), these observations need validation through further analyses owing to the limited sample size of this study.

Broadly, the suppression by LTA pretreatment was more evident for the case with the CpG/D-galN-treated mice. For example, IL-6 and TNF-*α* levels were reduced by LTA pretreatment. IFN-*γ* was also suppressed. Intriguingly, LTA pretreatment showed no or little suppression in R848/D-galN mice; for any of TNF-*α*, IL-6, and IFN-*γ*, there was no reduction relative to the mice without LTA pretreatment. These results demonstrate a discrepancy between the mortality data and cytokine data; although the LTA pretreatment improved the mortality for both combinations of CpG/D-galN and R848/D-galN, the tolerance-inducing effect of the LTA pretreatment on the levels of serum cytokines was clear for CpG/D-galN but not for R848/D-galN.

## 4. Concluding Discussion

The TLR/D-galN system has been widely used as a model of sepsis and hepatitis, and in particular, for the evaluation of potential drugs that might protect the liver from failure. This study aimed to expand on previous studies on this system in terms of types of TLRL, effects on production of cytokines, and effect of cross-tolerance induction by TLR2L pretreatment. The present results showed that besides LPS, poly (I:C), and CpG, which have been studied by several authors, LTA and R848 can also induce hepatic injury in D-galN-sensitized mice. For CpG/D-galN-treated mice, there was no appreciable level of involvement of endogenous TLR4Ls. We further found that LTA pretreatment can induce cross-tolerance and improve the survival rate against secondary treatment with either CpG/D-galN or R848/D-galN. Despite the finding that hepatic injury by both combinations was suppressed by LTA pretreatment, these combinations exhibited notable differences. Without LTA pretreatment, R848/D-galN showed a greater ability to induce systemic illness compared to CpG/D-galN. Furthermore, the former combination induced increased production of TNF-*α*, IL-6, and IFN-*γ*, and intriguingly these increases were not suppressed by LTA pretreatment.

In the tolerance induced by the LTA pretreatment, this pretreatment more efficiently improved the survival of CpG/D-galN-treated mice compared to R848/D-galN-treated mice (Figure 2). It was further noted that CpG/D-galN generally showed benign characteristics in that it did not induce systemic organ failure as R848/D-galN did in our setting. TLR9 appeared to be more liver specific, while TLR7/8 caused more systemic damage although hepatic injury was present. Although further comparison is beyond the scope of this study, the differential effects between these TLRLs may be discussed from a perspective on the relevance of the TLRL/D-galN system to sepsis or systemic inflammation. Although the original proposal that the LPS/D-galN system, similar to high-dose LPS treatment, could be considered as a septic model was questioned by several authors who showed that severe apoptotic liver injury was induced by LPS/D-galN [3] and poly (I:C)/D-galN [7], our findings raised a possibility that relatively uncharacterized TLRL/D-galN combinations may show more features of systemic inflammation than the LPS/D-galN system.

It is most likely that our LTA pretreatment elicited many negative regulatory mechanisms that have been elucidated in many studies, causing the tolerance against the second challenges in our setting. LPS tolerance has been extensively studied, but TLR2-induced tolerance has been shown to be important in some settings. Analyses by Turner et al. showed that *Brugia malayi* female worm- (BMFE-) mediated cross-tolerance to various TLR stimulations is dependent on TLR2 and MyD88 but not on TLR4 [25]. In splenic dendritic cells of zymosan- (TLR2 and dectin-1 agonist) treated mice, TLR2 stimulation mediates the inductions of retinaldehyde dehydrogenase type 2 (Raldh2) and IL-10 expression and stimulates Foxp3^+^ T_reg_ cells induction, suggesting a generally high propensity for tolerance induction after TLR2 signaling [26].

In the studies of TLR-mediated tolerance, a number of negative regulatory controllers have been proposed [27]. Distinct mechanisms are likely to be employed between TLR2- and 4-induced tolerance. For example, in the case of IL-1R-associated kinase (IRAK) that directly interact with MyD88, LTA-induced tolerance does not involve IRAK degradation, unlike the LPS-induced tolerance [28]. Nonetheless, in peptidoglycan- (PGN and TLR2 agonist) induced tolerant cells, IRAK1 kinase activity was inhibited and Nakayama et al. further showed that IRAK-M, a negative regulator of TLR signaling, is induced, likely inhibiting the IRAK1 recruitment to TLR/MyD88 complexes. For another example, kinase activity of IRAK4 is not required for the induction of LPS tolerance but contributes significantly to TLR2-elicited homo- and cross-tolerance [29].

On the other hand, many regulators are likely to be shared by TLR2 and other TLRs in tolerance induction. Using SOCS- (suppressor of cytokine signaling-) 1-deficient macrophages, Nakagawa et al. showed that SOCS-1 is necessary for tolerance to LPS induced by LPS, CpG DNA, or MALP-2 (TLR2/6 agonist) [30]. SOCS-1 exerts its negative regulation through enhancing ubiquitination of several molecules including Mal, which has been shown to be an adaptor protein relatively specific to TLR2 and TLR4 signaling. Upon stimulation of these TLRs, Mal becomes phosphorylated and then recognized by SOCS-1 that causes ubiquitination and degradation [31]. In the case of Tollip that ubiquitinates TLRs and also binds to IRAK1 (thus blocking IRAK4 recruitment), Tollip mRNA upregulation occurs by both LPS and LTA treatment in intestinal epithelial cells [32]. The ubiquitin-modifying enzyme A20 (also referred to as TNF*α*-induced protein 3) is induced by stimulations by multiple TLR ligands (TLR2, 3, 4, and 9) and has been implicated for terminations of TLR responses [33]. For the kinases MSK1 and MSK2 that are phosphorylated by p38 MAPK and Erk and exert many negative feedback mechanisms in TLR signaling including IL-10 gene upregulation, LTA and CpG DNA as well as LPS have been shown to trigger activation of their kinase activities [34] For CD200, a cell-surface glycoprotein whose binding to CD200R triggers diverse immune inhibitory mechanisms, a range of TLR agonists such as LPS, PGN, poly (I:C), and CpG DNA can induce CD200 mRNA in macrophages in a c-Rel-dependent manner [35]. Almeida et al. showed a requisite role for TLR2 in *Mycobacterium bovis* Bacillus Calmette-Guérin- (BCG-) mediated upregulation of peroxisome proliferator-activated receptor *γ* (PPARγ) in macrophages [36]. Besides its roles in lipid metabolism, PPAR*γ* exerts an anti-inflammatory response in murine and human macrophages, mainly through suppressing activities of transcriptional factors including AP-1, STAT-1, and NF-*κ*B [37].

MicroRNAs have also been shown to be important in TLR2-induced tolerance. miR-146a has been shown to suppress expression of TNF receptor-activated factor 6 (TRAF6) and IRAK1. Besides LPS-induced tolerance [38], bacterial lipoprotein (TLR2 agonist) treatment-induced tolerance has been shown to upregulate the expression of miR-146a in human THP-1 promonocytic cells [39]. In the case of miR-155, the ligands for TLR2, 3, 4, and 9 have been shown to induce expression in macrophages [40]. miR-155 is considered to fine-tune TLR signaling as it targets not only MyD88, TAB2, and IKK*ε* but also negative regulators involving SHP-1 and SOCS-1 [41]. Nahid et al. also showed that PGN stimulation caused rapid upregulation of miR-132 and miR-212 THP-1 monocytes and primary macrophages thereby downregulating IRAK4 and that this induction was induction relative to LPS-induced miR-146a induction [42]. It is plausible that many negative regulators including those not considered above are jointly playing important roles in the tolerance we observed in this study. The procedure used in the study may be useful for quick assessments of systemic effects of homo- and cross-tolerance induction by TLR stimulations.

Currently, we can only provide speculative discussions; however, the difference between CpG and R848 shown in Table 3 could be related to the finding that IL-10 levels were high after CpG/D-galN treatment relative to that after R848/D-galN treatment (Table 3). This difference between CpG and R848 may be related to the previous findings on these TLRLs that showed that CpG stimulation with CpG induced high levels of IL-10 production from macrophages [43]. The authors also showed that endogenous IL-10 suppresses IL-12p70 expression induced by CpG, suggesting that CpG signaling has intrinsic mechanisms for feedback that limit the degree of inflammation. It is also plausible that the observed difference between CpG and R848 may be partly caused by differential expression patterns of TLRs. TLR7/8 is not expressed at significant levels in the intestinal epithelium, while TLR9 is abundant [44]. Such differential expressions of TLRLs could contribute to the differential effects shown above. It should also be considered that in combination with TLR2 activation, the activation of either of TLR2, 4, or 9 is suppressed [45], while to our knowledge, there has been no such report on TLR7/8.

Incidentally, our analyses showed strain dependency. To our knowledge, between-strain differences in responses to TLRLs have not been well studied, especially for C3H mice. Yet, Liu et al. [46] showed that TLR9 mRNA levels were much higher in dendritic cells (DC) from naive C57BL/6 mice than in those from BALB/c mice. The expression levels of mRNAs for TLR2, 4, 5, and 6 were low in C57BL/6 DCs from the spleen. Although we have not examined C3H mice in this respect, this pattern of mRNAs appears consistent with our findings that showed high sensitivity of C57BL/6 mice to CpG. As another well-studied example, genetic background has also been shown to be important in the Th1/Th2 balance and septic response in mice [47, 48]. C57BL/6 and BALB/c mice are regarded as Th1- and Th2-dominant mouse strains, respectively [49]. Macrophages from BALB/c mice exhibit a limited IFN-*γ*-producing activity relative to those from C57BL/6 mice [50, 51]. TNF-*α* and IL-12 productions upon TLR2L and TLR4L stimulation in C57BL/6 mouse macrophages were more pronounced than in macrophages of BALB/c mice [52]. Possibly because of this difference, BALB/c mice were more vulnerable to CLP-induced lethality than C57BL/6 mice. Although to our knowledge, C57BL/6 and C3H mice have not been compared in detail, our findings indicated differences in terms of mortality and efficacy of tolerance induction, suggesting the presence of unrecognized genetic influences in several other processes involving TLR functions in vivo.

In summary, the present study showed that in addition to LPS and poly (I:C) and CpG, LTA and R848 can induce hepatic injury in D-galN-sensitized mice. Further, we confirmed that CpG/D-galN is not influenced by endogenous ligands for TLR4. Finally, we showed that CpG and R848 treatments exhibit distinct effects; both showed hepatic injury but distinct degrees of systemic organ failure. The tolerance induction by LTA pretreatment was evident for both TLRLs but clearer for CpG. These findings highlight the differences among TLRs. Our results also demonstrated the presence of strain dependency in both systemic effects and cytokine patterns. Nonetheless, the scale of our analysis was limited, and therefore further experiments will be necessary for a better understanding regarding these issues.

## Figures and Tables

**Table 1:** Serum ALT levels in each TLRL/D-galN-treated mouse. Mean ± SD is shown.

Treatment (per mouse)	*n*	Serum ALT levels (U/1)^1^	
		C57BL/6J	C3H
PBS	5	79.8 ± 11.4	45.8 ± 12.3
D-galN 20 mg	5	71.0 ± 24.6	66.4 ± 25.2
LPS 1 *μ*g	4	100.0 ± 16.4	96.3 ± 48.1
LPS 0.01 *μ*g/D-galN 20 mg	5	280.3 ± 163.5	114.3 ± 38.8
LPS 0.1 *μ*g/D-galN 20 mg	3	4830.0 ± 1429.0 (died at 5-6 h)	14713.3 ± 3131.5
LPS 1 *μ*g/D-galN 20 mg	3	3193.3 ± 1885.0 (died at 5-6 h)	13335.0 ± 5877.2 (died at 6–9 h)
LTA 100 *μ*g	4	83.8 ± 55.1	54.0 ± 36.8
LTA 50 *μ*g/D-galN 20 mg	5	544.2 ± 162.1	91.8 ± 51.8
LTA 100 *μ*g/D-galN 20 mg	5	2780.0 ± 1128.6	126.8 ± 52.6
R848 10 *μ*g	4	90.3 ± 37.6	51.8 ± 4.4
R848 1 *μ*g/D-galN 20 mg	5	165.4 ± 64.9	1301.2 ± 1031.7
R848 10 *μ*g/D-galN 20 mg	5	9307.0 ± 5112.5 (died at 6–9 h)	6622.0 ± 2851.1 (died at 7–9 h)
poly (I:C) 5 *μ*g	4	77.4 ± 20.5	63.0 ± 27.2
poly (I:C) 2.5 *μ*g/D-galN 20 mg	5	15005.0 ± 1653.1	307.6 ± 254.7
poly (I:C) 5 *μ*g/D-galN 20 mg	5	6486.0 ± 5779.0 (died at 5–10 h)	270.6 ± 249.5
CpG DNA 10 *μ*g	4	76.5 ± 24.4	41.7 ± 17.4
CpG DNA 10 *μ*g/D-galN 20 mg	5	90.4 ± 22.9	70.8 ± 16.9
CpG DNA 20 *μ*g/D-galN 20 mg	5	10021.7 ± 3927.3 (died at 9-10 h)	318.8 ± 163.6

^1^The reference range (mean ± SD) of ALT has been reported to be 27.7 ± 3.09 and 25.6 ± 12.0 for C57BL/6 and C3H, respectively [20].

**Table 2:** Effect of CpG (20 *μ*g), R848 (10 *μ*g), and LTA (100 *μ*g) in D-galN- (20 mg/mouse) sensitized mice. Mean ± SD is shown.

	Survival	ALT (U/l)	TNF-*α* (pg/ml)	IL-6 (pg/ml)
PBS (*n* = 5)				
C57BL/6	All survived by 10 h	63.5 ± 14.5	3.2 ± 0.5	5.2 ± 1.8
C3H	All survived by 10 h	52.3 ± 10.8	2.1 ± 0.3	4.3 ± 2.8
CpG/D-galN (*n* = 8)				
C57BL/6	Four of eight died within 10 h	18330.0 ± 5482.9	644.3 ± 187.7	1984.7 ± 771.4
C3H	Four of eight died within 10 h	267.7 ± 173.5	31.5 ± 43.6	105.2 ± 100.4
R848/D-galN (*n* = 8)				
C57BL/6	All eight died within 9 h	12675.7 ± 6365.7	337.6 ± 140.3	1029.4 ± 927.3
C3H	All eight died within 9 h	5071.2 ± 2417.6	686.0 ± 406.7	1657.0 ± 813.2
LTA/D-galN (*n* = 3)				
C57BL/6	All survived by 10 h	2536.7 ± 1346.0	63.0 ± 18.0	3208.0 ± 1046.3
C3H	All survived by 10 h	103.3 ± 35.6	8.2 ± 7.0	626.2 ± 290.9

**Table 3:** Serum levels of cytokines (pg/ml) in a set of experiments with C57BL/6 mice. Mean ± SD is shown.

Cytokine	Time	PBS	CpG/D-galN^†^		R848/D-galN^†^	
		(*n* = 7)	LTA− (*n* = 7)	LTA+ (*n* = 7)	LTA− (*n* = 7)	LTA+ (*n* = 7)
ALT (U/l)		68 ± 13 (10 h)	17875.7 ± 5156.2 (7.5–10 h)	202.4 ± 108.2^∗∗∗^ (10 h)	14737.5 ± 3894.0 (6–8.5 h)	10112.1 ± 5229.2 (8-10 h)

TNF-*α*	5 h	1.5 ± 0.3	22.7 ± 21.0	7.3 ± 0.5	15.5 ± 10.8	9.6 ± 3.4
	10 h	n.t.	39.8 ± 13.0	3.0 ± 0.0^∗∗∗^	Not available (death)	n.t.

IL-6	1 h	2.4 ± 1.6	1984.7 ± 771.4	67.7 ± 29.7^∗∗∗^	1029.4 ± 927.3	668.6 ± 481.5
	5 h	2.1 ± 0.8	1270.5 ± 1199.0	112.5 ± 51.4	297.7 ± 210.9	359.4 ± 294.2
	10 h	n.t.	4424.8^1^ ± 3718.7	33.2 ± 24.8^∗∗∗^	Not available (death)	n.t.

INF-*γ*	1 h	1.4 ± 0.6	1.3 ± 0.5	1.6 ± 1.2^∗^	1.1 ± 0.6	1.5 ± 0.5
	5 h	1.3 ± 0.7	89.5 ± 74.6	13.7 ± 5.4^∗^	52.8 ± 33.1	54.0 ± 9.4
	10 h	n.t.	128.5^1^ ± 147.2	6.5 ± 4.8^∗∗∗^	Not available (death)	n.t.

IL-10	1 h	n.t.	23.7 ± 18.6	6.2 ± 8.3^∗^	6.9 ± 4.3	2.7 ± 1.7
	5 h	n.t.	90.2 ± 93.1	63.2 ± 62.0	26.6 ± 10.8	6.4 ± 4.7^∗∗∗^
	10 h	n.t.	270.0^1^ ± 249.5	2.0 ± 0.0^∗∗∗^	Not available (death)	n.t.

IL-17A	1 h	n.t.	2.1 ± 1.1	2.4 ± 2.2	1.75 ± 1.0	2.0 ± 1.3
	5 h	n.t.	2.6 ± 2.1	4.9 ± 3.5	1.1 ± 0.8	2.7 ± 0.8^∗∗∗^
	10 h	n.t.	1.0^1^ ± 0.6	1.0 ± 0.0	Not available (death)	n.t.

IL-23	1 h	n.t.	16.7 ± 3.4	10.5 ± 3.7^∗∗^	12.4 ± 4.7	14.3 ± 7.4
	5 h	n.t.	15.0 ± 4.6	16.5 ± 3.7	9.4 ± 3.8	16.7 ± 3.4 ^∗∗∗^
	10 h	n.t.	18.3^1^ ± 8.3	9.0 ± 0.0^∗^	Not available (death)	n.t.

IL-27	1 h	n.t.	4.6 ± 2.5	7.5 ± 5.0	2.6 ± 1.4	4.7 ± 2.6
	5 h	n.t.	8.2 ± 4.6	11.0 ± 8.2	3.1 ± 1.5	10.3 ± 5.6^∗∗∗^
	10 h	n.t.	5.5^1^ ± 3.0	2.4 ± 1.1^∗∗^	Not available (death)	n.t.

n.t.: not tested. ^†^PBS (LTA− mice) or 200 *μ*g/mouse LTA (LTA+ mice) was administered 24 h prior to the challenge with the 20 *μ*g CpG (or 10 *μ*g R848)/20 *μ*g D-galN. Asterisks indicate a significant difference between with and without the LTA pretreatment (^∗^*p* < 0.05, ^∗∗^*p* < 0.03, and ^∗∗∗^*p* < 0.01). ^1^Two out of seven mice died within 10 h, and the five mice that survived within 10 h were analyzed.

## References

[1] Galanos C., Freudenberg M. A., Reutter W. (1979). Galactosamine-induced sensitization to the lethal effects of endotoxin. *Proceedings of the National Academy of Sciences of the United States of America*.

[2] Jiang W., Sun R., Wei H., Tian Z. (2005). Toll-like receptor 3 ligand attenuates LPS-induced liver injury by down-regulation of toll-like receptor 4 expression on macrophages. *Proceedings of the National Academy of Sciences of the United States of America*.

[3] Mignon A., Rouquet N., Fabre M. (1999). LPS challenge in D-galactosamine-sensitized mice accounts for caspase-dependent fulminant hepatitis, not for septic shock. *American Journal of Respiratory and Critical Care Medicine*.

[4] Nowak M., Gaines G. C., Rosenberg J. (2000). LPS-induced liver injury in D-galactosamine-sensitized mice requires secreted TNF-α and the TNF-p55 receptor. *American Journal of Physiology - Regulatory, Integrative and Comparative Physiology*.

[5] Stachlewitz R. F., Seabra V., Brandford B. (1999). Glycine and uridine prevent D-galactosamine hepatotoxicity in the rat: role of Kupffer cells. *Hepatology*.

[6] Yi A. K., Yoon H., Park J. E., Kim B. S., Kim H. J., Martinez-Hernandez A. (2006). CpG DNA-mediated induction of acute liver injury in D-galactosamine-sensitized mice: the mitochondrial apoptotic pathway-dependent death of hepatocytes. *Journal Biological Chemistry*.

[7] Dejager L., Libert C. (2008). Tumor necrosis factor alpha mediates the lethal hepatotoxic effects of poly(I:C) in d-galactosamine-sensitized mice. *Cytokine*.

[8] Dunne A., Marshall N. A., Mills K. H. (2011). TLR based therapeutics. *Current Opinion in Pharmacology*.

[9] Teixeira-Coelho M., Cruz A., Carmona J. (2011). TLR2 deficiency by compromising p19 (IL-23) expression limits Th17 cell responses to mycobacterium tuberculosis. *International Immunology*.

[10] Lima C. X., Souza D. G., Amaral F. A. (2015). Therapeutic effects of treatment with anti-TLR2 and anti-TLR4 monoclonal antibodies in polymicrobial sepsis. *PLoS One*.

[11] Gao J., Jiang Z., Wang S., Zhou Y., Shi X., Feng M. (2016). Endoplasmic reticulum stress of Kupffer cells involved in the conversion of natural regulatory T cells to Th17 cells in liver ischemia-reperfusion injury. *Journal of Gastroenterology and Hepatology*.

[12] Shimamoto A., Chong A. J., Yada M. (2006). Inhibition of Toll-like receptor 4 with eritoran attenuates myocardial ischemia-reperfusion injury. *Circulation*.

[13] Anderberg S. B., Luther T., Frithiof R. (2016). Physiological aspects of Toll-like receptor 4 activation in sepsis-induced acute kidney injury. *Acta Physiologica*.

[14] Duthie M. S., Windish H. P., Fox C. B., Reed S. G. (2011). Use of defined TLR ligands as adjuvants within human vaccines. *Immunological Reviews*.

[15] Trinchieri G., Sher A. (2007). Cooperation of Toll-like receptor signals in innate immune defense. *Nature Reviews Immunology*.

[16] Anuradha R., Chakraborty K., Ray P. (2013). Immunosuppressive MDSCs induced by TLR signaling during infection and role in resolution of inflammation. *Frontiers in Cellular and Infection Microbiology*.

[17] Dobrovolskaia M. A., Medvedev A. E., Thomas K. E. (2003). Induction of in vitro reprogramming by toll-like receptor (TLR)2 and TLR4 agonists in murine macrophages: effects of TLR “homotolerance” versus “heterotolerance” on NF-κB signaling pathway components. *The Journal of Immunology*.

[18] Arshad M. I., Patrat-Delon S., Piquet-Pellorce C. (2013). Pathogenic mouse hepatitis virus or poly(I:C) induce IL-33 in hepatocytes in murine models of hepatitis. *PLoS One*.

[19] Kuroda N., Inoue K., Ikeda T., Hara Y., Wake K., Sato T. (2014). Apoptotic response through a high mobility box 1 protein-dependent mechanism in LPS/GalN-induced mouse liver failure and glycyrrhizin-mediated inhibition. *PLoS One*.

[20] Charles river (2017). Control data of each strain. http://www.crj.co.jp/cms/pdf/info_common/17/6033572/Contro_data_6_April_2011ca.pdf.

[21] Bianch M. E. (2007). DAMPs, PAMPs and alarmins: all we need to know about danger. *Journal of Leukocyte Biology*.

[22] Jacinto R., Hartung T., McCall C., Li L. (2002). Lipopolysaccharide- and lipoteichoic acid-induced tolerance and cross-tolerance: distinct alterations in IL-1 receptor-associated kinase. *The Journal of Immunology*.

[23] Sato S., Nomura F., Kawai T. (2000). Synergy and cross-tolerance between toll-like receptor (TLR) 2- and TLR4-mediated signaling pathways. *The Journal of Immunology*.

[24] Furuya S., Kono H., Hara M., Hirayama K., Sun C., Fujii H. (2015). Interleukin 17A plays a role in lipopolysaccharide/d-galactosamine-induced fulminant hepatic injury in mice. *Journal of Surgical Research*.

[25] Turner J. D., Langley R. S., Johnston K. L. (2006). *Wolbachia* endosymbiotic bacteria of *Brugia malayi* mediate macrophage tolerance to TLR- and CD40-specific stimuli in a MyD88/TLR2-dependent manner. *The Journal of Immunology*.

[26] Manicassamy S., Ravindran R., Deng J. (2009). Toll-like receptor 2-dependent induction of vitamin A-metabolizing enzymes in dendritic cells promotes T regulatory responses and inhibits autoimmunity. *Nature Medicine*.

[27] Kondo T., Kawai T., Akira S. (2012). Dissecting negative regulation of Toll-like receptor signaling. *Trends in Immunology*.

[28] Nakayama K., Okugawa S., Yanagimoto S. (2004). Involvement of IRAK-M in peptidoglycan-induced tolerance in macrophages. *Journal of Biological Chemistry*.

[29] Xiong Y., Pennini M., Vogel S. N., Medvedev A. E. (2013). IRAK4 kinase activity is not required for induction of endotoxin tolerance but contributes to TLR2-mediated tolerance. *Journal of Leukocyto Biology*.

[30] Nakagawa R., Naka T., Tsutsui H. (2002). SOCS-1 participates in negative regulation of LPS responses. *Immunity*.

[31] Mansell A., Smith R., Doyle S. L. (2006). Suppressor of cytokine signaling 1 negatively regulates Toll-like receptor signaling by mediating Mal degradation. *Nature Immunology*.

[32] Otte J. M., Cario E., Podolsky D. K. (2004). Mechanisms of cross hyporesponsiveness to Toll-like receptor bacterial ligands in intestinal epithelial cells. *Gastroenterology*.

[33] Boone D. L., Turer E. E., Lee E. G. (2004). The ubiquitin-modifying enzyme A20 is required for termination of Toll-like receptor responses. *Nature Immunology*.

[34] Ananieva O., Darragh J., Johansen C. (2008). The kinases MSK1 and MSK2 act as negative regulators of Toll-like receptor signaling. *Nature Immunology*.

[35] Mukhopadhyay S., Plüddemann A., Hoe J. C. (2010). Immune inhibitory ligand CD200 induction by TLRs and NLRs limits macrophage activation to protect the host from meningococcal septicemia. *Cell Host & Microbe*.

[36] Almeida P. E., Silva A. R., Maya-Monteiro C. M. (2009). *Mycobacterium bovis* Bacillus Calmette-Guérin infection induces TLR2-dependent peroxisome proliferator-activated receptor γ expression and activation: functions in inflammation, lipid metabolism, and pathogenesis. *The Journal of Immunology*.

[37] Seki R., Nishizawa K. (2016). Factors regulating Th17 cells: a review. *Biomedical Research and Clinical Practice*.

[38] Nahid M. A., Pauley K. M., Satoh M., Chan E. K. (2009). miR-146a is critical for endotoxin-induced tolerance: IMPLICATION IN INNATE IMMUNITY. *The Journal of Biological Chemistry*.

[39] Quinn E. M., Wang J. H., O'Callaghan G., Redmond H. P. (2013). MicroRNA-146a is upregulated by and negatively regulates TLR2 signaling. *PLoS One*.

[40] O'Connell R. M., Taganov K. D., Boldin M. P., Cheng G., Baltimore D. (2007). MicroRNA-155 is induced during the macrophage inflammatory response. *Proceedings of the National Academy of Sciences of the United States of America*.

[41] Li Y., Shi X. (2013). MicroRNAs in the regulation of TLR and RIG-I pathways. *Cellular and Molecular Immunology*.

[42] Nahid M. A., Yao B., Dominguez-Gutierrez P. R., Kesavalu L., Satoh M., Chan E. K. (2013). Regulation of TLR2-mediated tolerance and cross-tolerance through IRAK4 modulation by miR-132 and miR-212. *The Journal of Immunology*.

[43] Boonstra A., Rajsbaum R., Holman M. (2006). Macrophages and myeloid dendritic cells, but not plasmacytoid dendritic cells, produce IL-10 in response to MyD88- and TRIF-dependent TLR signals, and TLR-independent signals. *The Journal of Immunology*.

[44] Lee J., Mo J. H., Katakura K. (2006). Maintenance of colonic homeostasis by distinctive apical TLR9 signaling in intestinal epithelial cells. *Nature Cell Biology*.

[45] Dalpke A. H., Lehner M. D., Hartung T., Heeg K. (2005). Differential effects of CpG-DNA in toll-like receptor-2/-4/-9 tolerance and cross-tolerance. *Immunology*.

[46] Liu T., Matsuguchi T., Tsuboiet N., Yajima T., Yoshikai Y. (2002). Differences in expression of Toll-like receptors and their reactivities in dendritic cells in BALB/c and C57BL/6 mice. *Infection and Immunity*.

[47] Stewart D., Fulton W. B., Wilson C. (2002). Genetic contribution to the septic response in a mouse model. *Shock*.

[48] Hsieh C. S., Macatonia S. E., O'Garra A., Murphy K. M. (1995). T cell genetic background determines default T helper phenotype development in vitro. *The Journal of Experimental Medicine*.

[49] Mills C. D., Kincaid K., Alt J. M., Heilman M. J., Hill A. M. (2000). M-1/M-2 macrophages and the Th1/Th2 paradigm. *The Journal of Immunology*.

[50] Kuroda E., Yamashita U. (2003). Mechanisms of enhanced macrophage-mediated prostaglandin E2 production and its suppressive role in Th1 activation in Th2-dominant BALB/c mice. *The Journal of Immunology*.

[51] Kuroda E., Kito T., Yamashita U. (2002). Reduced expression of STAT4 and IFN-γ in macrophages from BALB/c mice. *The Journal of Immunology*.

[52] Watanabe H., Numata K., Ito T., Takagi K., Matsukawa A. (2004). Innate immune response in Th1- and Th2-dominant mouse strains. *Shock*.

